# Dynamic cultivation of human mesenchymal stem/stromal cells for the production of extracellular vesicles in a 3D bioreactor system

**DOI:** 10.1007/s10529-024-03465-4

**Published:** 2024-02-13

**Authors:** Ciarra Almeria, René Weiss, Maike Keck, Viktoria Weber, Cornelia Kasper, Dominik Egger

**Affiliations:** 1 Institute of Cell and Tissue Culture Technology, Department of Biotechnology, University of Natural Resources and Life Sciences, Vienna, Austria; 2 Center for Biomedical Technology, Department for Biomedical Research, University for Continuing Education Krems, Krems, Austria; 3 Department of Plastic, Reconstructive and Aesthetic Surgery, Agaplesion Diakonieklinikum Hamburg, Hamburg, Germany; 4 Institute of Cell Biology and Biophysics, Leibniz University Hannover, Hannover, Germany

**Keywords:** 3D cell culture, Bioreactors, Extracellular vesicles, Hypoxia, Mesenchymal stem cells

## Abstract

**Purpose:**

3D cell culture and hypoxia have been demonstrated to increase the therapeutic effects of mesenchymal stem/stromal cells (MSCs)-derived extracellular vesicles (EVs). In this study, a process for the production of MSC-EVs in a novel 3D bioreactor system under normoxic and hypoxic conditions was established and the resulting EVs were characterized.

**Methods:**

Human adipose-derived MSCs were seeded and cultured on a 3D membrane in the VITVO® bioreactor system for 7 days. Afterwards, MSC-EVs were isolated and characterized via fluorescence nanoparticle tracking analysis, flow cytometry with staining against annexin V (Anx5) as a marker for EVs exposing phosphatidylserine, as well as CD73 and CD90 as MSC surface markers.

**Results:**

Cultivation of MSC in the VITVO® bioreactor system demonstrated a higher concentration of MSC-EVs from the 3D bioreactor (9.1 × 10^9^ ± 1.5 × 10^9^ and 9.7 × 10^9^ ± 3.1 × 10^9^ particles/mL) compared to static 2D culture (4.2 × 10^9^ ± 7.5 × 10^8^ and 3.9 × 10^9^ ± 3.0 × 10^8^ particles/mL) under normoxic and hypoxic conditions, respectively. Also, the particle-to-protein ratio as a measure for the purity of EVs increased from 3.3 × 10^7^ ± 1.1 × 10^7^ particles/µg protein in 2D to 1.6 × 10^8^ ± 8.3 × 10^6^ particles/µg protein in 3D. Total MSC-EVs as well as CD73^−^CD90^+^ MSC-EVs were elevated in 2D normoxic conditions. The EV concentration and size did not differ significantly between normoxic and hypoxic conditions.

**Conclusion:**

The production of MSC-EVs in a 3D bioreactor system under hypoxic conditions resulted in increased EV concentration and purity. This system could be especially useful in screening culture conditions for the production of 3D-derived MSC-EVs.

**Supplementary Information:**

The online version contains supplementary material available at 10.1007/s10529-024-03465-4.

## Introduction

2D culture on tissue culture treated polystyrene surfaces, such as petri dishes, well-plates or T-flasks, do not resemble the native environment of mesenchymal stem/stromal cells (MSCs). Remarkably, prolonged culture on these surfaces was shown to impair therapeutically relevant cell properties, to lead to genetic instability, and to increases the risk for malignant transformation of MSCs (Wang et al. 2013; Capelli et al. 2014; Hladik et al. 2019). In contrast, 3D cell culture systems were shown to increase the therapeutic potential or maintain the stem cell characteristics of MSCs (Cheng et al. 2013; Guo et al. 2014; Ylostalo et al. 2017; Kouroupis and Correa 2021). This is especially important for the production of MSCs and MSC-derived extracellular vesicles (EVs) for cell-therapy applications. EVs are cell-derived membranous particles acting as signaling molecules for communication between cells (Camussi et al. 2013). They transmit information of their parental cells by delivering cargo, which influences both, physiological and pathological processes in recipient cells (Lai et al. 2011; Maumus et al. 2020). EVs are released by cells of the body and consist of three subclasses of different biogenesis: apoptotic bodies (> 1000 nm), microvesicles (100 to 1000 nm), and exosomes (40 to 100 nm) (György et al. 2011; Witwer et al. 2019). However, the generic term extracellular vesicles is used to describe both exosomes and microvesicles, as these subgroups overlap in size and cannot be discriminated with the available technology to date (Théry et al. 2018). Different effects on recipient cells have been attributed to the cargo and origin of EVs (Tkach and Théry 2016; Tsiapalis and O’Driscoll 2020). EVs derived from MSCs (Rani et al. 2015) have attracted particular attention, as they exhibit beneficial effects in immunomodulation (Song et al. 2020), induction of apoptosis of T-cells (Li et al. 2019), induction of angiogenesis (Kang et al. 2016) and in promoting the proliferation of various cell types (Fan et al. 2020). In this regard, the use of 3D culture systems during MSC-EVs production resulted in increased EV secretion (Haraszti et al. 2018) and pronounced therapeutic properties, such as immunomodulation (Kim et al. 2020; Cao et al. 2020) or enhanced osteogenesis in comparison to 2D MSC-EVs (Yu et al. 2022).

As a result of rapidly increasing research towards MSC-EVs, awareness towards cell culture parameters including oxygen concentration, 3D culture, pH, starvation, and cytokine pre-conditioning, has grown, as environmental conditions affect the composition and release kinetics of EVs (Almeria et al. 2022). For example, Patel et al. investigated the influence of different culture parameters and concluded that the biological activity of MSC-derived EVs differs depending on the cell source and culture parameters, such as medium composition, oxygen concentration, duration of culture, as well as shear stress (Patel et al. 2017). Oxygen levels in the tissues of the human body are below 21% O_2_ and thus tissue-specific oxygen concentrations, although described as “hypoxic”, might be “normoxic” from a cellular perspective. However, in the following, we refer to hypoxia with regards to the atmospheric oxygen level of 21% O_2_. Hypoxic preconditioning (1–10% O_2_) of MSCs was reported to significantly enhance the proliferative activity, generate distinctive modifications in stem cell properties, and influence the secretion of cytokines and growth factors compared to cultivation under normoxic conditions at 21% O_2_ (Kinnaird et al. 2004; Nekanti et al. 2010). Also, the cargo and biological functionalities of MSC-EVs were found to be affected by hypoxic preconditioning of the parental cells (Gonzalez-King et al. 2017; Konoshenko et al. 2018; Almeria et al. 2019; Mao et al. 2022; Huang et al. 2022; Zhou et al. 2022).

Therefore, bioreactors that tightly monitor and control the cellular microenvironment are increasingly important for the production of EVs. So far, mainly stirred tank bioreactors in combination with microcarrier culture have been investigated for the production of therapeutic doses of EVs (Cao et al. 2020; de Almeida Fuzeta et al. 2020). However, microcarrier cultures do not represent true 3D culture systems, as cells are forced to grow on an almost planar 2D surface. In contrast, the VITVO® presents a small-scale perfusion bioreactor with a fiber-based matrix which allows for true 3D culture of MSCs.

Therefore, we investigated whether the VITVO® system represents a suitable 3D bioreactor for the production of MSC-EVs in a hypoxic environment. For this, we established a process for the expansion of human adipose-derived MSCs under normoxic (21% O_2_) and hypoxic (5% O_2_) conditions in the 3D bioreactor system and compared it to a standard 2D static EV production process. We analyzed the MSC-EVs harvested from both systems by (fluorescence) nanoparticle tracking analysis (NTA) and flow cytometry. In the future, this bioreactor system could be used to screen, compare and understand the impact of process cell culture parameters on the biological functionalities of MSC-EVs which would help to optimize MSC-EV production towards specific therapeutic applications.

## Materials and methods

### Cell culture

The use of human tissue from abdominoplasties was approved by the ethics committee of the University of Lübeck (Reference number 20-333, November 2020), and the donor (female, 75 years old) gave written consent. Human MSCs were isolated within 8 h after surgery as previously described (Egger et al. 2017). MSCs were cultivated in standard culture medium composed of MEM alpha (Thermo Fisher Scientific, Waltham, MA, USA), 0.5% gentamycin (Lonza, Basel, Switzerland), 2.5% human platelet lysate (HPL; PL BioScience, Aachen, Germany; filtered through 0.2 μm filters) and 1 IU/mL heparin (Ratiopharm, Ulm, Germany) in a humidified atmosphere at 37 °C, 5% CO_2_ and 21% or 5% O_2_, and cryo-preserved in liquid nitrogen. The HPL used in the culture medium was EV-depleted by centrifugation at 120,000 × g at 4 °C for 16 h before use. Upon use, cryopreserved human adipose-derived mesenchymal stem cells (MSCs) were thawed and subcultivated to passage 2. Briefly, the cryovial was transferred to a 37 °C water bath and thawed. Subsequently, 1 mL room temperature (RT) alpha-MEM basal medium (Thermo Fisher Scientific) was added, the cell suspension was transferred to a centrifugation tube and the vial was rinsed with 2 mL RT alpha-MEM. Afterwards, the cell suspension was filled up to 10 mL with RT MEM alpha and centrifuged for 5 min at 300 × g. The supernatant was removed and cells were resuspended in 15 mL pre-warmed standard culture medium. Then, the cell suspension was transferred to a T-75 flask and incubated at 37 °C, 5% CO_2_ under normoxic (21% O_2_) or hypoxic (5% O_2_) conditions. To detach cells for further cultivation they were rinsed with phosphate-buffered saline (PBS; Thermo Fisher Scientific) and incubated in accutase (Sigma Aldrich) at 37 °C. Accutase treatment was quenched with a 1.5-fold volume of culture medium and the cell suspension was transferred into a centrifuge tube for centrifugation at 300 × g for 5 min. After the removal of the supernatant, the pellet was resuspended in a culture medium, and the cell number was determined with a hemocytometer. For subcultivation, cells were seeded at 3000 cells/cm^2^ and allowed to grow 48 h before harvest.

### Seeding and bioreactor culture

VITVO® (Rigenerand, Modena, Italy) is a small and portable bioreactor developed to provide a 3D tissue-like matrix in a closed system. The bioreactor consists of a perimetral frame TPE (thermoplastic elastomer) with two optical transparent oxygenation membranes, allowing for continuous gas exchange and visibility during cell cultivation. Furthermore, the fiber-based matrix is composed of an inert and biocompatible synthetic polyester with a thickness of 400 μm and provides 4 cm^2^ of surface area. The randomly distributed fibers have a diameter of 1.7 μm and the bioreactor’s empty volume which is 1.4 mL represents approximately 90% of the entire volume (Candini et al. 2019).

Before use, the bioreactor system was rinsed several times with a basal medium to remove potential membrane residues from the manufacturing process. The system was filled (1.4 ± 0.2 mL) with basal medium for 1 h at 37 °C, 5% CO_2,_ and 21% (normoxic) or 5% O_2_ (hypoxic), to equilibrate the membrane to the cultivation conditions. Subsequently, the VITVO® was seeded with 1 × 10^6^ MSCs (0.25 × 10^6^/cm^2^) as described before (Candini et al. 2019). The bioreactor was closed and incubated at 37 °C and 5% CO_2_ under normoxic or hypoxic conditions for at least 6 h to allow cells to adhere to the membrane before the start of perfusion.

**Fig. 1:**
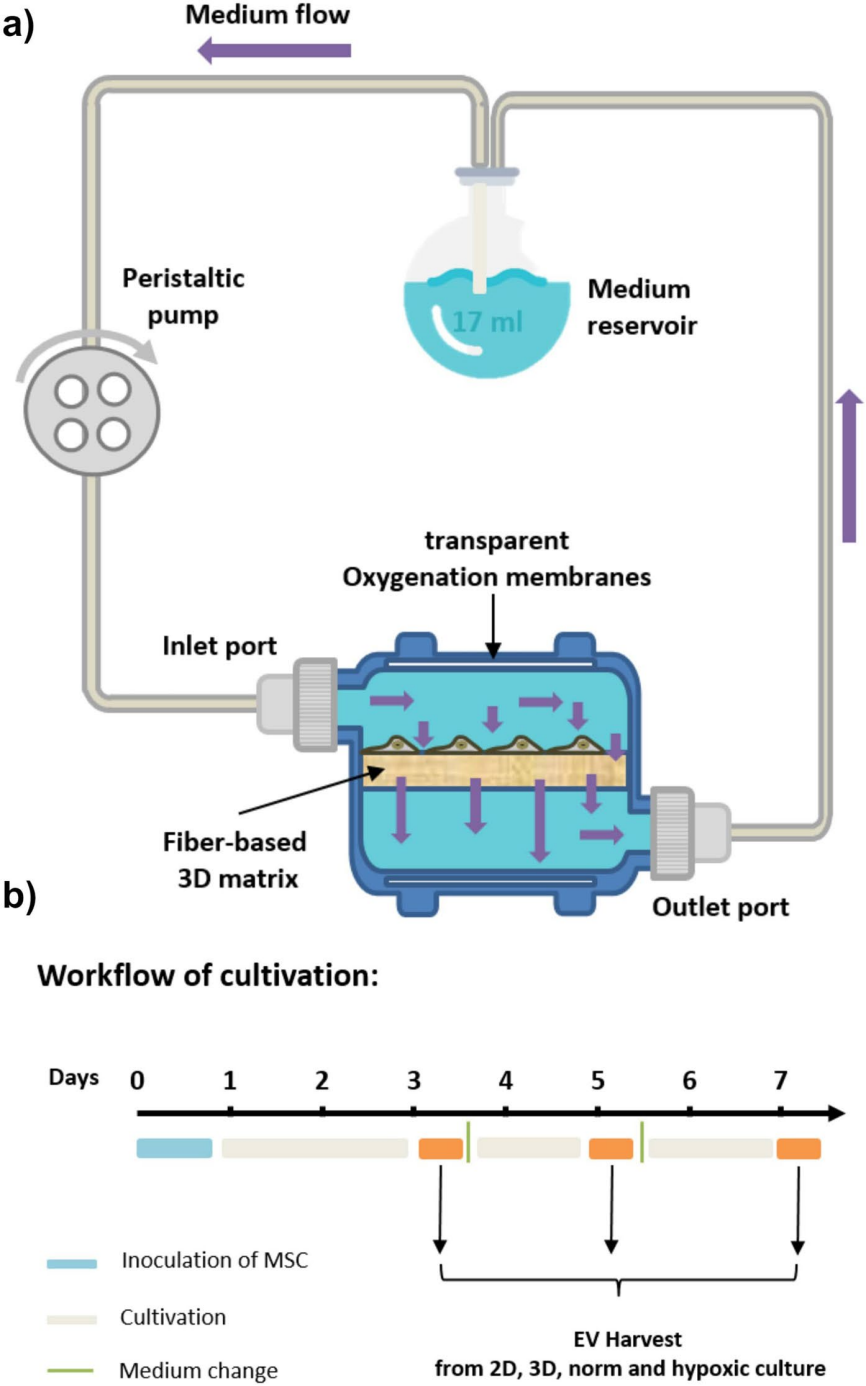
Scheme of the 3D cell culture setting in vitro. **a** The 3D bioreactor system VITVO® was connected to a perfusion pump in order to generate dynamic conditions during mesenchymal stem/stromal cell (MSC) cultivation. Flow rate was set to 1 mL/min in order to provide cells with an exchange of fresh nutrients from the cell culture medium as well as constant waste removal. **b** Workflow of cell culture regime performed for the cell culture and production as well as the harvest of MSC-derived extracellular vesicles

Afterwards, the bioreactor was connected to a closed tubing system (Fig. 1a) including a medium reservoir containing 17 mL of EV-depleted culture medium (alpha-MEM, 2.5% HPL (pre-processed by ultracentrifugation at 120,000 × g at 4 °C for 16 h), 0.5% Gentamycin (10 mg/mL), 1 U/mL Heparin). The perfusion with a peristaltic pump (ISMATEC, Opfikon, Switzerland) was set to 1 mL/min. As a control, MSCs were also cultured in a 2D system. For this purpose, 0.25 × 10^6^/cm^2^ cells were seeded in T-75 flasks with 17 mL of the same medium used for 3D cultivation at 37 °C, 5% CO_2,_ and normoxic or hypoxic conditions. The cells were cultured for 7 days, and the complete medium was exchanged at days 3, 5, and 7 and stored at −20 °C for further analyses (Fig. 1b). Additionally, 1 mL of conditioned medium from the medium reservoir was sampled each day for glucose and lactate measurements using the YSI 2900 Biochemistry Analyzer (Xylem Inc., OH, USA). At the end of the bioreactor cultivation, cells were stained with calcein acetoxymethyl ester (calcein-AM, Sigma Aldrich) and incubated for 16 h. Images were obtained at excitation/emission wavelengths of 490/515 nm with a fluorescence microscope (DM IL LED by Leica, Wetzlar, Germany).

### Isolation of extracellular vesicles from cell culture medium

MSCs were cultured in 2D and 3D systems as described above. Culture supernatants from days 3, 5, and 7 were thawed, pooled and centrifuged at 500 × g for 5 min and at 1500 × g for 15 min at RT to remove cells and debris. The resulting supernatant was subjected to an additional centrifugation step at 100,000 × g, 4 °C for 90 min using a Sorvall WX 80 ultracentrifuge with SW 32 Ti rotor (Beckman Coulter Inc., CA, US). The resulting pellet was resuspended in 0.3 mL PBS without Mg^2+^ and Ca^2+^ (Thermo Fisher Scientific) and stored at − 20 °C until further characterization.

### Nanoparticle tracking analysis of MSC-EVs

EV size distribution profiles and concentration measurements were obtained by nanoparticle tracking analysis (NTA; Zeta View, Particle Metrix, Inning, Germany). Silica 100 nm microspheres (Polysciences, Inc., Warrington, PA, US) were routinely analyzed to check instrument performance (Gardiner et al. 2013). Measurements were performed at RT using the following instrument settings: 80 (sensitivity), 1000 (maximal area), 5 (minimal area), and 25 (brightness). These settings were established for light scatter mode (LSM) using silica 100 nm microspheres and subsequently adjusted for optimal detection of MSC-EVs. MSC-EV suspensions were diluted in 0.3 mL of (1X) PBS UltraPure™ (Thermo Fisher Scientific, Waltham, MA, USA). The acquisition temperature was maintained at 20 °C. Each sample was recorded 10 times for 30 s, using fresh samples for each acquisition. The detection chamber was thoroughly washed with PBS between each sample measurement.

Additionally, particle tracking analyses in fluorescence mode (FM) were performed with a 488 nm laser and a 500 nm long-pass filter for fluorescence detection. Device calibration for fluorescence measurements was performed using the OR520 standard (100 nm, Particle Metrix). 30 µL of MSC-EV suspensions were stained with 5 µL cell mask green (CMG) dye stock solution (Invitrogen, MA, USA) for 3 h at 37 °C in the dark. Afterwards, all samples were diluted 1:50 with PBS UltraPure to provide a concentration of 1 × 10^8^–1 × 10^9^ particles/mL. In addition, controls consisting of PBS UltraPure were prepared and stained in the same manner to detect possible CMG aggregation during sample preparation that could distort the measurements. All counts were performed in triplicates for each sample. Fluorescence measurements were performed at RT in a dark room, to minimize photobleaching of labeled samples. The following instrument settings were used for FM: 90 (sensitivity), 300 (shutter), 1000 (maximal area), 5 (minimal area), 7 (trace length), and 15 (brightness). A threshold level of 7 was applied for video processing. Data were acquired by video recording of one cycle of measurement over 11 positions and were analyzed using the software ZetaView version 8.04.02 (Particle Metrix, Inning, Germany).

### The protein content of MSC-derived EVs

Protein concentration was measured by the micro bicinchoninic acid (BCA) assay (Thermo Scientific). Briefly, according to the manufacturer’s instructions, 150 µL EVs or the blank standard were pipetted into a microplate well (n = 3) and 150 µL of working reagent was added. Then, the plate was put on a plate shaker for about 30 s prior to incubation at 37 °C for 2 h. Afterwards, the plate was allowed to cool down to RT before absorbance at 562 nm was measured on a plate reader (Infinite M1000 Pro, TECAN, Männerdorf Switzerland).

### Flow cytometric characterization of MSCs

To characterize MSCs that were used for EV production, 100 µL of cell suspension (1 × 10^6^/mL) was stained with anti-CD73 PE (0.3 µg/mL) and anti-CD90 APC AF750 (1 µg/mL; all from Beckman Coulter) to detect the MSC surface markers ecto-5′-nucleotidase and Thy-1, respectively. Staining was performed for 15 min in the dark. Stained samples were diluted fivefold in PBS and analyzed on a CytoFlex-LX flow cytometer (Beckman Coulter) equipped with 375 nm, 405 nm, 488 nm, 561 nm, and 638 nm lasers. Data were acquired for 3 min at a flow rate of 30 μL/min and analyzed using Kaluza 2.1 (Beckman Coulter).

### Flow cytometric characterization of MSC-derived EVs

To characterize MSC-derived EVs, EV suspensions isolated as described above were diluted 1:100 in 0.1 µm filtered AnnexinV (Anx5) binding buffer (ABB) and stained with APC-conjugated Anx5 (0.1 µg/mL; Becton Dickinson) as marker for phosphatidylserine (PS) as well as with anti-CD73 PE (0.6 µg/mL) and anti-CD90 APC-AF750 (2.5 µg/mL; both from Beckman Coulter). To remove any precipitates, fluorochrome conjugates were centrifuged at 18,000 × g for 10 min at 4 °C before use. Stained samples were diluted fivefold in ABB and analyzed on a CytoFlex-LX flow cytometer. Fluorescent-green silica particles (1.0 µm, 0.5 µm, 0.1 µm; excitation/emission 485/510 nm; Kisker Biotech, Steinfurt, Germany) were used for calibration, the triggering signal was set to violet side scatter (405 nm), and the EV gate was set below the 1 µm bead cloud as previously described (Tripisciano et al. 2017; Weiss et al. 2018). Data were acquired for 2 min at a flow rate of 10 μL/min and analyzed using Kaluza 2.1 (Beckman Coulter). To calculate the number of events for each sample, the dilution factor during sample preparation and staining was considered. MSC-EVs were defined as Anx5^+^ events in the EV gate.

### Statistical analysis

Statistical analysis was performed using GraphPad Prism, version 8.2 (La Jolla, CA, USA). Data are presented as mean ± standard deviation. Unpaired t-test was used for the determination of differences between two groups. Nonparametric unpaired one-way ANOVA test (Kruscal-Wallis test) with Dunn’s multiple comparisons test was used for the determination of differences between more than two groups. Significance is indicated as follows: *p < 0.05, **p < 0.01, ***p < 0.001, ****p < 0.0001.

## Results

### MSC expansion and medium conditioning for MSC-EV production

**Fig. 2:**
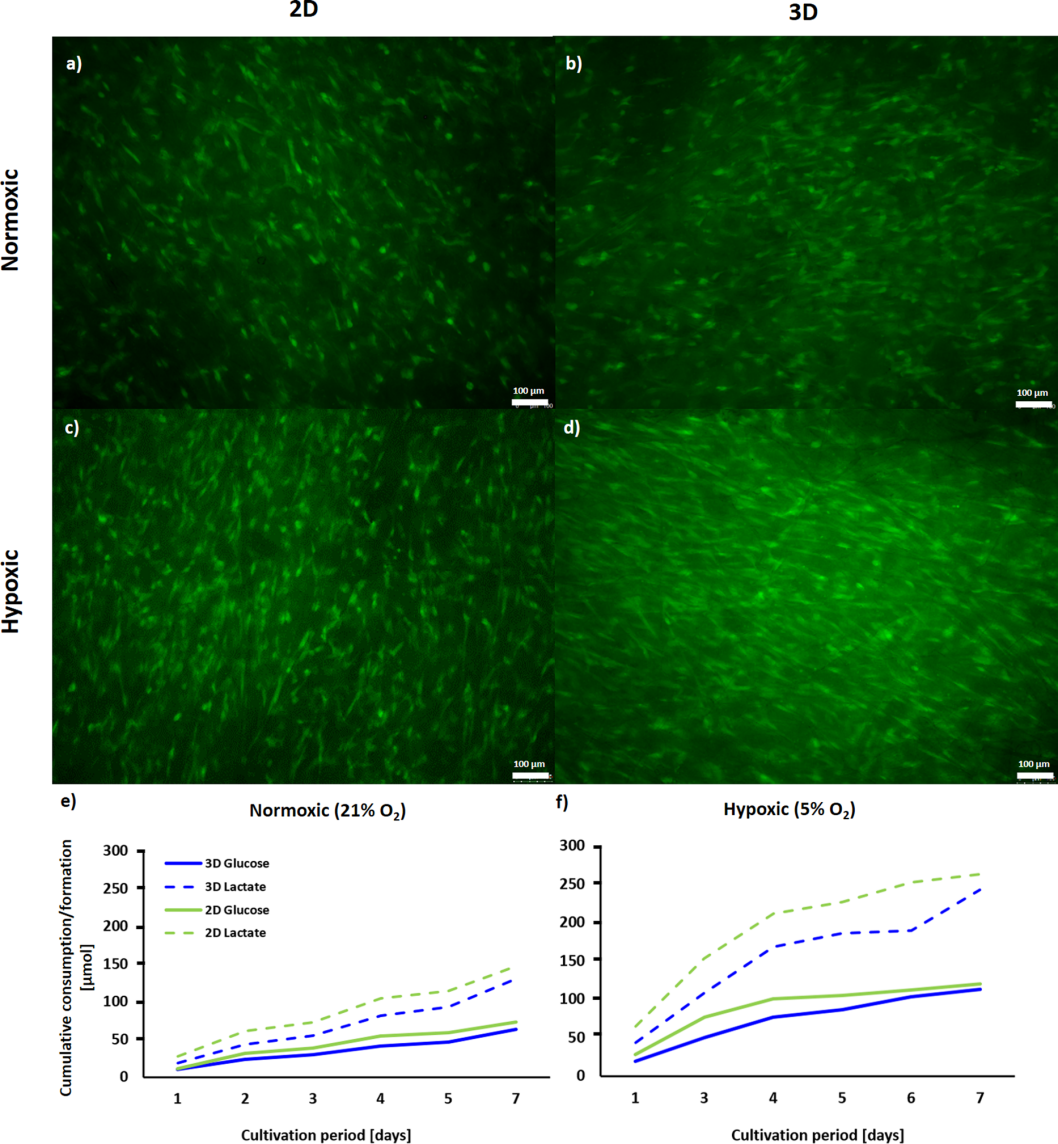
Cell viability and course of glucose consumption and lactate production of MSCs. Cells were cultivated in 2D static or 3D VITVO® bioreactor system under normoxic (21% O_2_) or hypoxic (5% O_2_) conditions. **a**–**d** Calcein-AM viability staining, as well as **e** monitoring of glucose consumption and **f** lactate production, were performed to verify cell viability and metabolic activity of MSCs, respectively. Data is represented as mean (n = 3). Scale bar = 100 µm

In this work, a bioreactor culture system with a fiber-based matrix was used for the cultivation of MSCs in 3D. Most MSCs developed a typical spindle-like morphology as indicated by viability staining (Fig. 2). As it was not possible to retrieve cells from the matrix, we could not account for non-adherent cells that might have adhered during the seeding process. Assuming a similar seeding density, the glucose consumption and lactate production in 2D static and 3D bioreactor culture was similar in both, normoxic and hypoxic conditions. However, MSCs consumed significantly more glucose and produced significantly more lactate under hypoxic conditions in 2D static and in 3D bioreactor culture on the last day of culture (as indicated by unpaired t-test; Fig. 2e, f).

### Size distribution of MSC-EVs

**Fig. 3:**
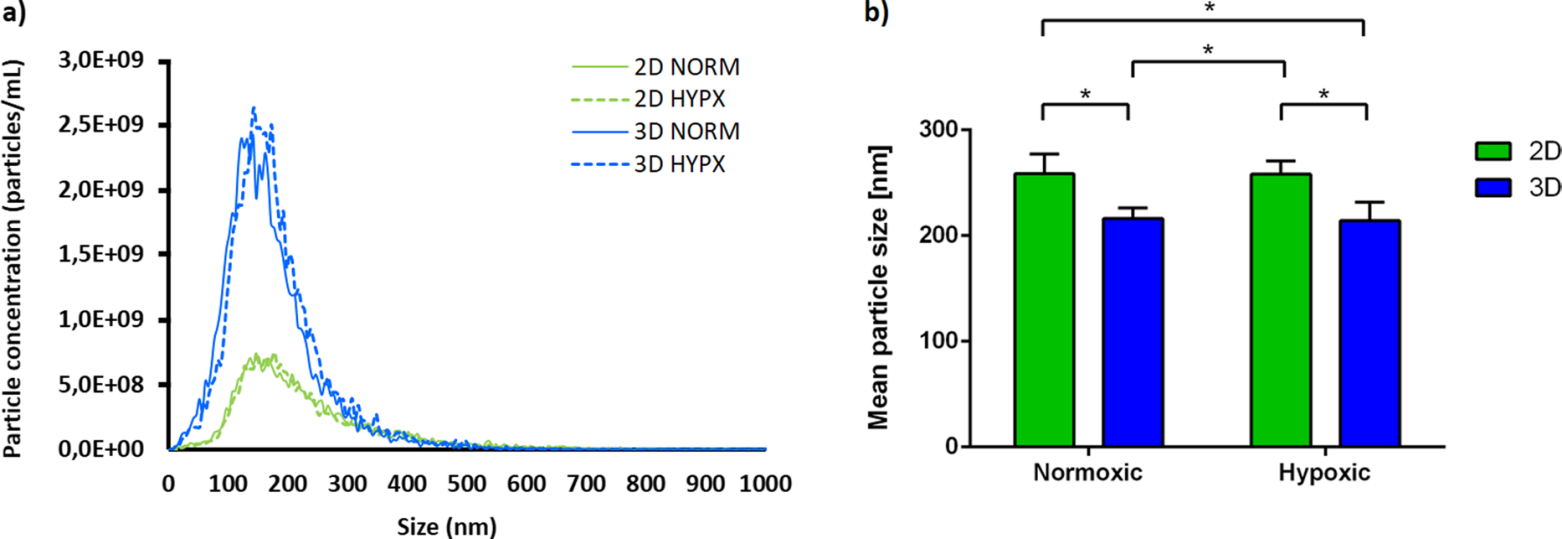
Size distribution and mean size of MSC-derived EVs. The characterization was performed using nanoparticle tracking analysis (NTA) in scatter mode as described in Materials and Methods. **a** Particle distribution and **b** mean particle size of MSC-derived EVs from 2D static and 3D VITVO® bioreactor cultivation under normoxic or hypoxic conditions from cumulative collection of EV samples from days 3, 5 and 7. Data are presented as mean ± SD (n = 3; *p < 0.05)

To assess differences between EVs secreted in the 2D and VITVO® conditions, the EVs were isolated from conditioned media and collected after 7 days using serial ultracentrifugation. Representative particle size distributions confirmed that 2D and 3D MSC-EVs had their peak at a particle size of < 200 nm (Fig. 3a) which is the expected range for EVs isolated from the cell culture supernatant. No significant difference was observed between normoxic and hypoxic conditions. The mean average size of EVs produced in 2D static was significantly larger, possibly due to vesicle aggregation or fusion (Fig. 3b).

### Purity of MSC-EVs

**Fig. 4:**
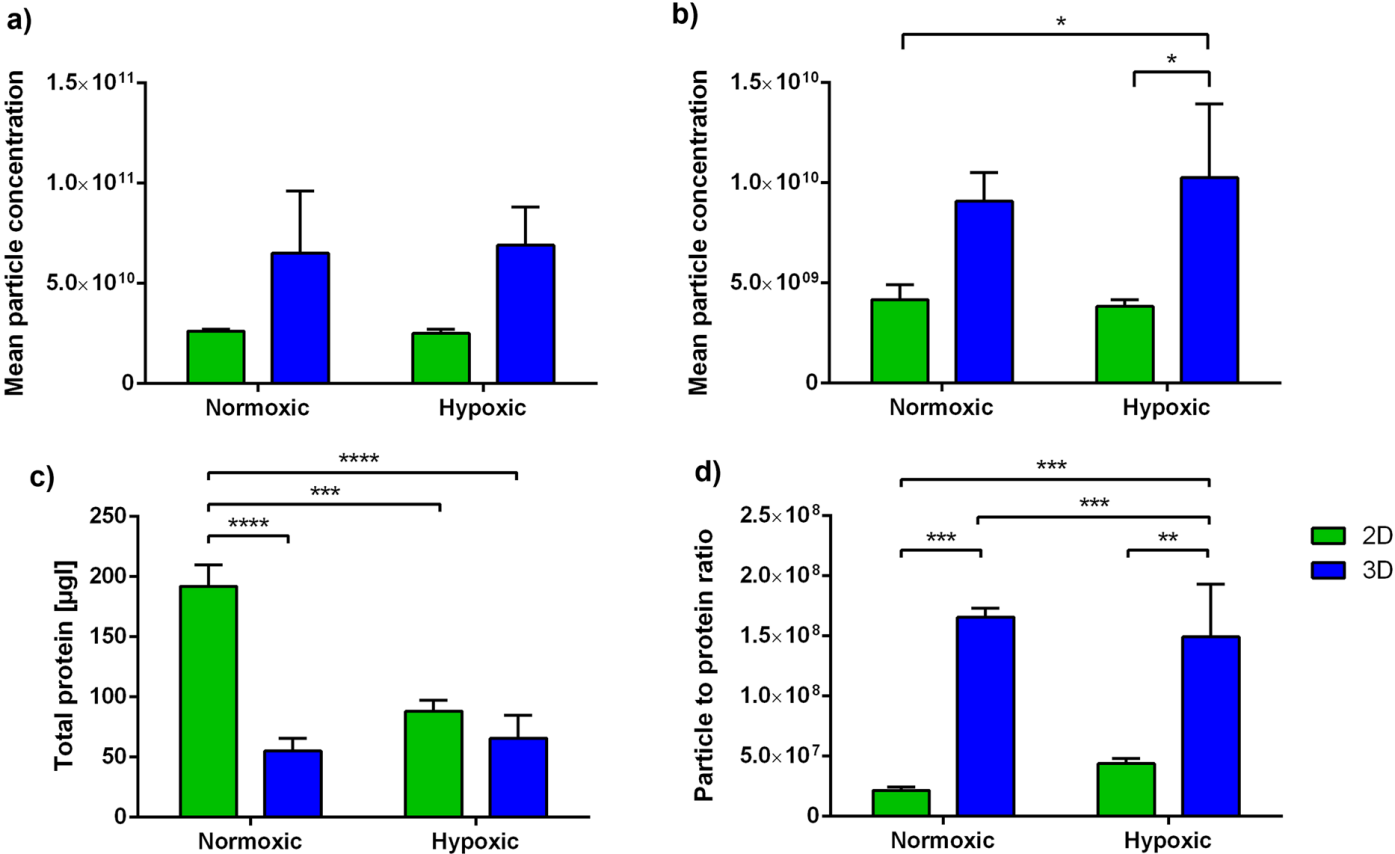
Characterization of MSC-derived EVs from 2D and 3D VITVO® bioreactor cultivation under normoxic and hypoxic conditions. Mean particle concentration of MSC-EVs measured by nanoparticle tracking analysis (NTA) in **a** scatter and **b** fluorescence mode stained with cell mask green (CMG) dye. **c** Total protein was determined by bicinchoninic acid (BCA) protein assay to further calculate the** d** particle-to-protein ratio (PPR). Data are represented as mean ± SD (n = 3; *p < 0.05; **p < 0.01; ***p < 0.001; ****p < 0.0001)

Quantification of the EV yield was performed by NTA after EV isolation and compared across the different culture conditions. NTA in scatter mode showed no significant differences in particle concentrations (Fig. 4a). However, fluorescence NTA revealed a substantial difference (p < 0.001) between 2 and 3D cultivation, with 3D having a higher average particle concentration (9.4 × 10^9^ ± 2.3 × 10^8^ particles/mL) compared to 2D (4.0 × 10^9^ ± 5.2 × 10^8^ particles/mL) (Fig. 4b). Both, normoxic and hypoxic conditions in 3D also showed a 1.5-fold increase in particle concentration compared to 2D MSC-EVs.

The total protein of MSC-EVs obtained from 2D and 3D under hypoxic conditions did not reveal significant differences (Fig. 4c). Nevertheless, MSC-EVs from 2D normoxic cell culture indicate significantly higher protein content as compared to 3D. A particle-to-protein ratio (PPR) was determined to assess the purity of EV samples by dividing the particle concentration (determined by NTA) by the total protein concentration in the same sample (Fig. 4d). The higher the PPR, the lower the amount of co-isolated protein contaminants, hence, the higher the sample purity (Webber and Clayton 2013). Overall, the PPR was relatively constant in the VITVO bioreactor system, with an average of 1.6 × 10^8^ ± 8.3 × 10^6^ particles/µg protein for hypoxic and normoxic conditions and revealed high purity compared to 2D isolated MSC-EVs.

### Surface marker analysis

**Fig. 5:**
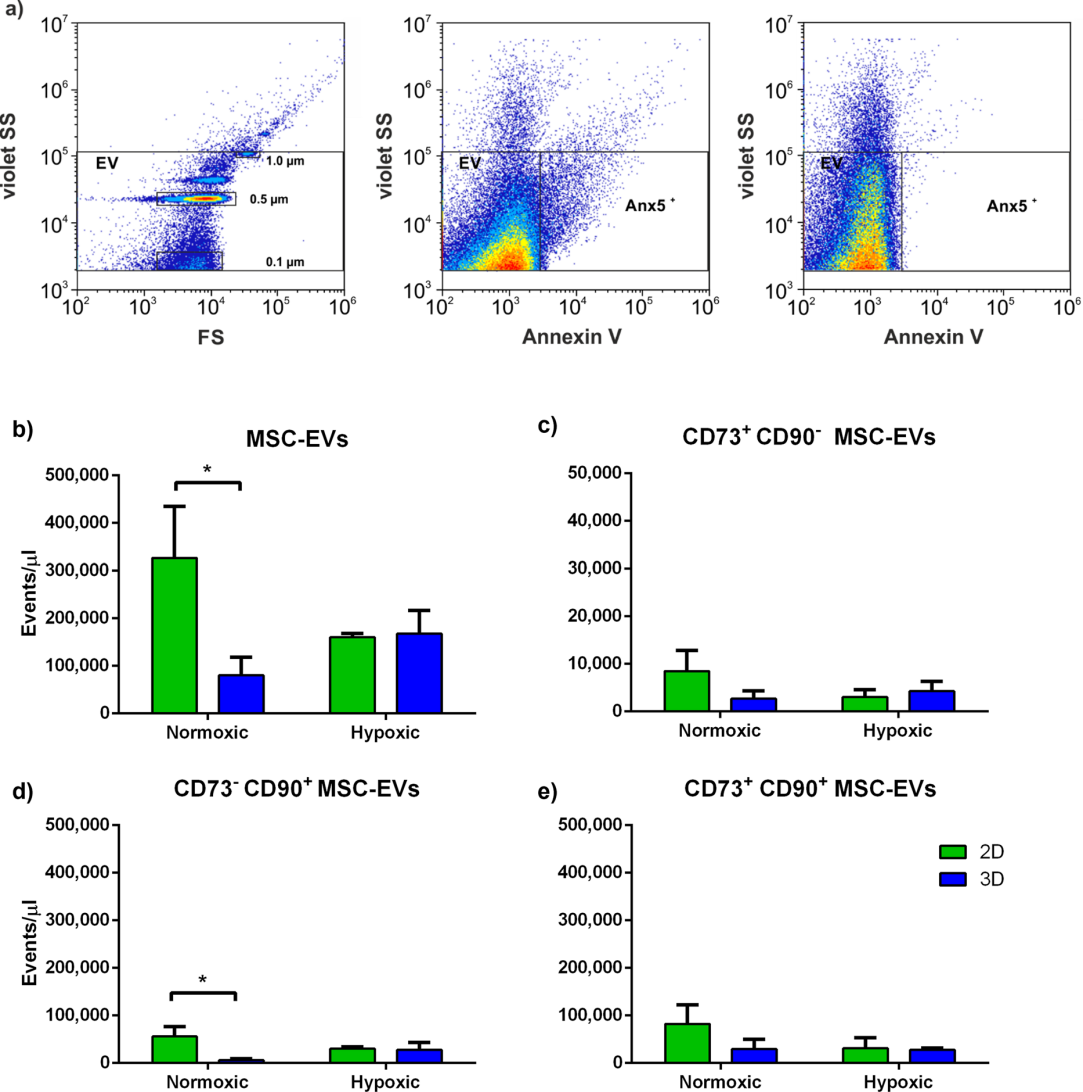
Characterization of EVs using flow cytometry. **a** Flow cytometric calibration was performed with fluorescent-green silica particles (1.0, 0.5, and 0.1 µm), and the EV gate was set at the 1 µm bead cloud (left panel). EV suspensions were stained with APC-conjugated annexin V (Anx5) as marker of phosphatidylserine (PS) as well as anti-CD73-FITC-PE and anti-CD90 APC-AF750 as MSC surface markers. A side scatter vs. annexin V (SS vs. Anx5) dot plot for EV suspension is shown as example (middle panel). **b** MSC-EVs were defined as Anx^+^ events in the EV gate. Amounts of **c** CD73^+^CD90^−^, **d** CD73^−^CD90^+^, and **e** CD73^+^CD90^+^ MSC-EVs are shown. Data are given as mean ± SD (n = 3; *p < 0.05)

The expression of MSC surface markers CD73 and CD90 on MSCs was determined via flow cytometry to test the functionality of used antibodies. According to flow cytometric analysis, 96% of all MSCs were positive for CD73 and CD90 (Supplementary Fig. S1, Fig. 5a). For the analysis of EVs from MSCs cultivated under normoxic or hypoxic conditions in 2D static and 3D bioreactor culture, we observed a significantly higher amount of MSC-EVs from 2D compared to 3D culture (Fig. 5b). While, the number of CD73^+^CD90^−^ MSC-EVs did not differ in any culture condition (Fig. 5c), CD73^−^CD90^+^ MSC-EVs were significantly higher in 2D culture compared to 3D culture under normoxic conditions (Fig. 5d). Overall, CD73^+^CD90^+^ MSC-EVs indicate no statistical significance (p < 0.05) between all four culture groups (Fig. 5e).

## Discussion

Three-dimensional (3D) cell culture is an emerging field that provides a more physiological environment for the expansion and differentiation of MSCs, compared to conventional monolayer systems. The quality of 3D cell culture models and their ability to represent the behavior of cells in in vivo tissues is critical to producing relevant data in regenerative medicine. Therefore, extensive research has been conducted over the years to investigate and develop various 3D cell culture systems not only for MSCs but cells generally used for in vitro models. Cell therapy approaches using MSCs have been recognized as efficient using different disease models (Ullah et al. 2015; Zhao and Liu 2016; Zhou et al. 2021; Kronstadt et al. 2022). Numerous studies have provided evidence that the secretion of EVs mediates the therapeutic effects of MSCs through paracrine mechanisms (Kaur Sarhadi et al. 2021; Hassanzadeh et al. 2021; Murali and Holmes 2021; Racchetti and Meldolesi 2021). EVs enable cellular communication by transferring proteins, metabolites, and nucleic acids to recipient cells (Loussouarn et al. 2021; Liu et al. 2021; Gupta et al. 2022). EVs lack a nucleus and are not able to self-replicate, which eliminates intrinsic tumorigenic risks. The small size of EVs enables filter sterilization, and handling and storage of EVs are easier compared to MSCs. Therefore, EV-based therapeutics offer several advantages over direct cellular approaches, resulting in an increasing number of translational studies involving MSC-EVs. MSC-EVs may serve as an alternative approach to cell-based therapies, which could potentially minimize adverse effects caused by MSCs (Van Niel et al. 2018; Yin et al. 2019; Mendt et al. 2019). Nevertheless, the lack of consistent results remains challenging in developing and translating EVs for therapeutic applications.

This study aimed to establish a process for the production of MSC-EVs from human adipose-derived MSCs under normoxic (21% O_2_) and hypoxic (5% O_2_) conditions in a 3D culture bioreactor system and to compare it to a standard 2D static EV production process. Following a successful expansion of MSCs in both cell culture platforms, EVs were collected from the conditioned medium and were characterized by NTA for particle size and concentration. We found that MSC-EV concentrations increased in the 3D bioreactor (1.5 ± 0.5-fold increase). As it was not possible to detach the cells from the matrix, to assess the seeding efficiency or the cell number at later time points of the culture process, it is not clear whether this increase is caused by a higher cell number or a higher EV secretion per cell in 3D. In another study, human MSCs were expanded on xeno-free microcarriers (SoloHill, PALL) in a Vertical Wheel bioreactor (PBS 0.1 MAG bioreactor, PBS Biotech Inc., CA, US) with a working volume of 100 mL. The authors obtained 5.1 ± 2.1 × 10^9^ particles/mL on average, from an initial MSC number of 5 × 10^6^ cells and 2 g of microcarriers (de Almeida Fuzeta et al. 2020). These results are comparable to the average concentration (10.0 ± 3.1 × 10^9^ particles/mL) observed in our study, resulting from only 1 × 10^6^ cells. This increase in EV secretion in the VITVO bioreactor could have been stimulated by the applied fluid flow in the system, as similar correlations have already been described in previous studies upon mechanical stimulation for EV production (Morrell et al. 2018; Guo et al. 2021; Chen et al. 2021; Kusuma et al. 2022; Kronstadt et al. 2023). However, despite reports demonstrating enhanced EV release by MSCs, when cultured under hypoxic conditions ranging from 0.1–3% O_2_, compared to 10–21% O_2_ (Ge et al. 2021; Chen et al. 2021), both in our previous comparative study in 2D (Almeria et al. 2019) as well as in this present work, no significant correlation in EV productivity and oxygen tension have been observed. Nevertheless, all of these factors might have contributed to the increase of EV productivity in the VITVO bioreactor and reveals the potential of 3D cell culture as compared to traditional cell culture platforms. However, additional studies would be needed to separately determine the contribution of each of these parameters on cellular processes.

Additionally, EV purity was assessed by estimating the PPR for each EV sample. PPR was elevated in MSC-EVs from 3D bioreactor compared to those produced under 2D static conditions (Fig. 4). An increase in PPR, when MSC-EVs were produced in a perfusion bioreactor, was observed before (Kronstadt et al. 2023). Constant medium flow results in more homogeneous access of the cells to nutrients, thus allowing a more robust MSC-EV manufacturing process. Therefore, the VITVO bioreactor platform described in this study could allow a robust production of MSC-EVs at higher purities, compared to static culture systems.

Furthermore, we investigated the differences in expression of several EV and MSC surface markers of EVs derived from 2 and 3D conditions by flow cytometry. We observed differences regarding the EV concentration when comparing NTA and flow cytometric analysis. This could be explained by the different minimum detectable vesicle sizes for NTA (~ 50 nm) vs. flow cytometry (~ 150 nm) and the detection of non-EV light scattering structures, such as protein aggregates or lipoproteins, by NTA. This could explain the lower EV counts detected in flow cytometry as compared to NTA measurements in our study since the majority of EVs in our study have a size of ≤ 150 nm. Furthermore, NTA in fluorescence mode demonstrated that only 16% of all particles detected in scatter mode were stained with CMG, a fluorescent membrane dye (Nielsen et al. 2018), indicating that NTA detects a high proportion of non-membranous light-scattering structures. The findings from flow cytometry and NTA analyses suggest that EVs from 2D are larger in size than EVs from this specific 3D culture. Other papers have found EVs from 2D to be similar or larger in size (Zhang et al. 2021; Yuan et al. 2022). These inconsistencies might be due to the 3D culture system used and the used method to determine the size.

The analysis of the surface markers by flow cytometry indicated also that throughout all conditions only a fraction of MSC-EVs were positive for CD73 and CD90. This could be due to the ultracentrifugation step in the isolation process, which may cause damage of epitopes, ultimately resulting in decreased antibody binding. Milder isolation procedures such as size exclusion chromatography could be used to avoid possible damages of the epitopes.

Finally, we would like to discuss the limitations of our study. While this proof-of-concept study provides valuable insights, it is important to note that the results may have a more widespread applicability when considering donor diversity, including both sex and age, as well as tissue source of MSCs. To draw broader conclusions, it would be beneficial to explore MSC-EVs from multiple donors and various tissue types. Furthermore, despite our focus on measuring the quantity and purity of EVs, it is vital to recognize that biological functions including migration, immune regulation, cell proliferation, and angiogenesis, are mainly caused by proteins and RNAs encapsulated within EVs, which were not evaluated. Furthermore, it was not possible to assess the initial cell number or the cell number at later stages of the culture. In this context, it should be noted that due to proliferation of cells, the flow through the matrix might have been restricted at some point in culture which might have further affected growth dynamics and gene expression. Thus, it remains unclear whether the observed increase of EVs in 3D compared to 2D is due to higher EV secretion per cell or due to a higher cell number. Therefore, future studies with this system will focus on assessing EV production kinetics in dependence on process parameters such as the flow rate and medium composition. Another focus will be assessing the cargo and biological functions utilizing suitable potency assays to assess angiogenic and immunomodulatory properties of MSC-EVs from 3D and hypoxic conditions.

Overall, our findings highlight a successful culture of MSCs and the production of MSC-EVs in a 3D bioreactor system under normoxic and hypoxic conditions. No significant effects of the oxygen concentration on EV quantity, purity, or surface marker expression were observed. However, the quantity and purity of MSC-EVs were significantly elevated using the 3D bioreactor, compared to 2D static culture. Further studies are required to assess the effect of these culture parameters on the functional therapeutic properties of EVs. This culture platform holds great potential for parallel miniaturized cultures in a physiologic (3D, hypoxic) environment, still allowing for rapid screening of culture conditions, such as flow rates, oxygen concentrations, cell density, or even co-cultures to optimize EV production processes towards a specific therapeutic application.

### Supplementary Information

Below is the link to the electronic supplementary material.

Supplementary file1 (DOCX 264 KB)

## Data Availability

The datasets used and/or analyzed during the current study are available from the corresponding author on reasonable request.
