# Supplementary figures and images for: Dynamic cultivation of human mesenchymal stem/stromal cells for the production of extracellular vesicles in a 3D bioreactor system
Source: Biotechnol Lett. 2024 Feb 13;46(2):279–93. doi: 10.1007/s10529-024-03465-4 (PMC10902030; doi:10.1007/s10529-024-03465-4)

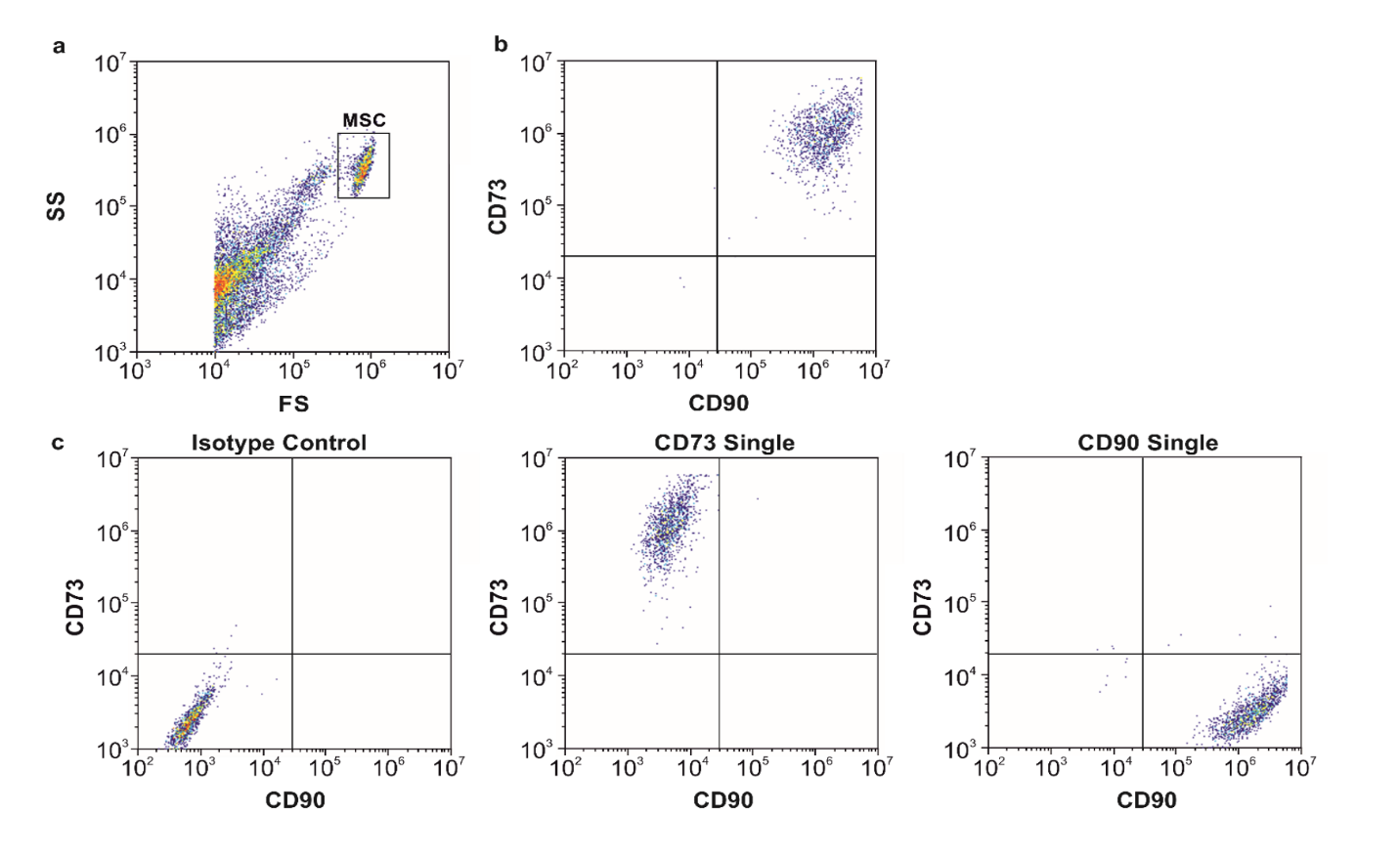
 Supplementary Figure 1: Surface marker analysis of MSCs

Supplement: Supplementary file 1 — Supplementary file1 (DOCX 264 KB) [file 10529_2024_3465_MOESM1_ESM.docx]
